# Supplementary material for: Design, Characterization, and Anticancer and Antimicrobial Activities of Mucoadhesive Oral Patches Loaded with Usnea barbata (L.) F. H. Wigg Ethanol Extract F-UBE-HPMC
Source: Antioxidants (Basel). 2022 Sep 13;11(9):1801. doi: 10.3390/antiox11091801 (PMC9495557; doi:10.3390/antiox11091801)
Supplement: Supplementary file 1 [file antioxidants-11-01801-s001.zip › Figures S1 and S2.pdf]

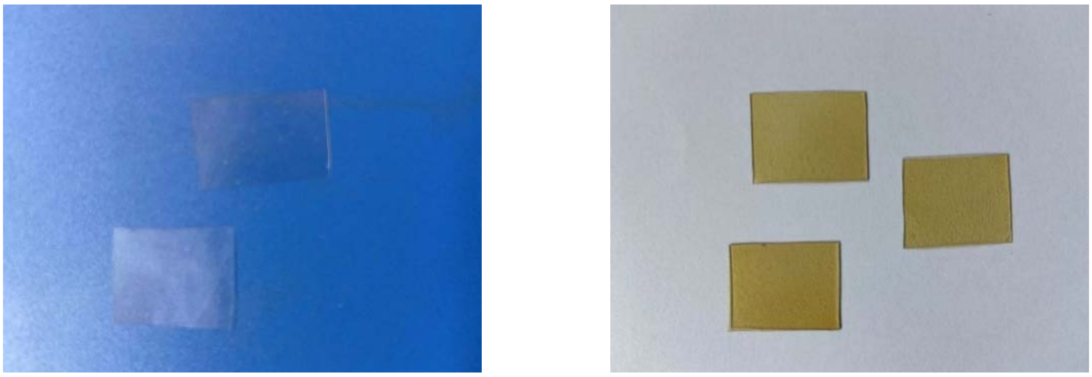

(a) (b)

Figure S1. Mucoadhesive oral patches: (a) Reference, (b) F-UBE-HPMC

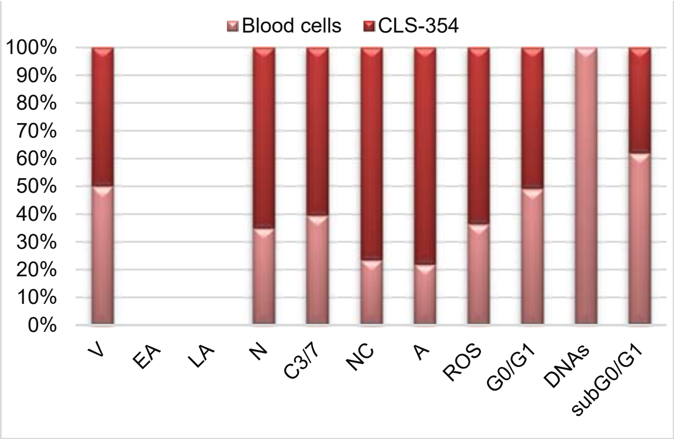

A

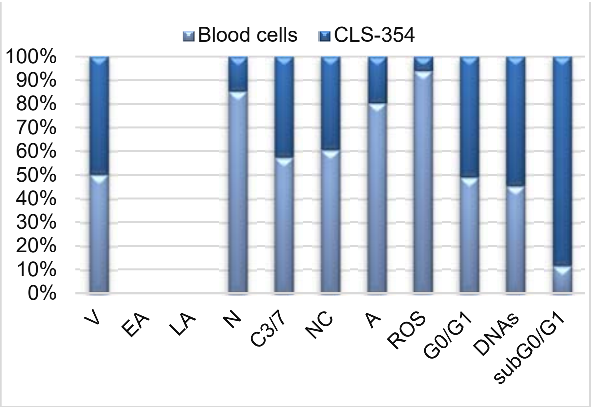

B

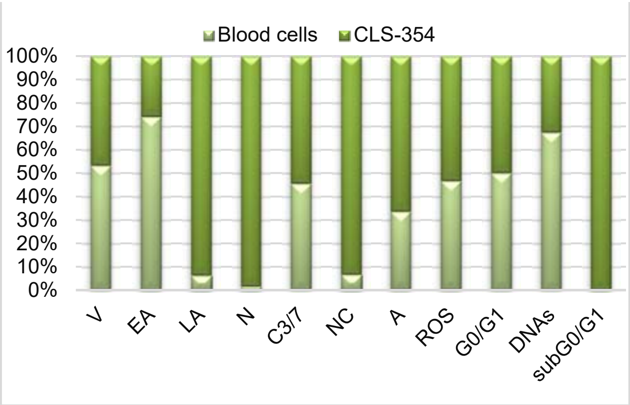

C

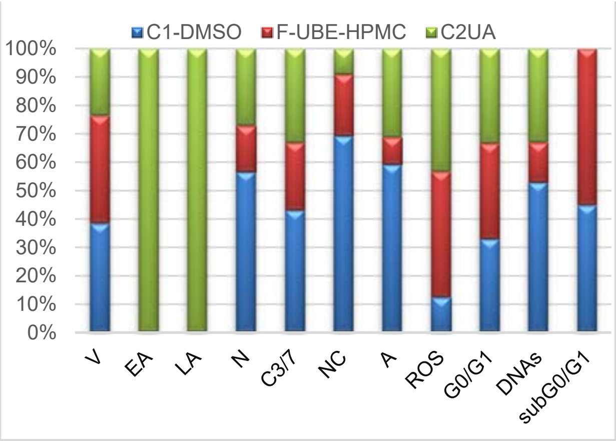

D

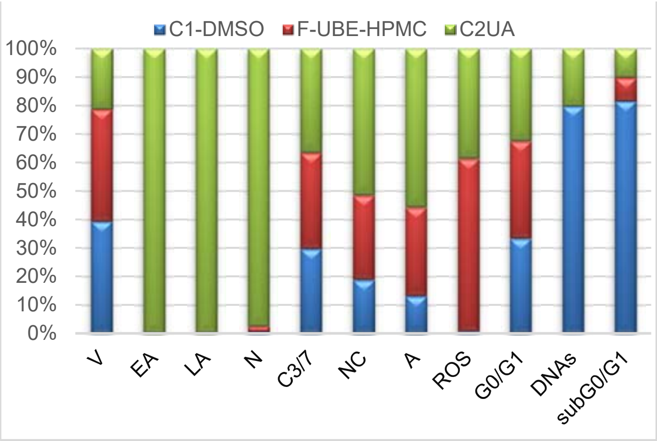

E

Figure S2. Comparative activity on normal blood cells and CLS-354 tumor cells of F-UBE-HPMC and both controls. A. F-UBE-HPMC; B. 1% DMSO (negative control); C. UA of 125 μg/mL (positive control); D,E. An overview of F-UBE-HPMC films' activity and controls on normal blood cells (D) and OSCC tumor cells (E). V—viability, EA—early apoptosis, LA—late apoptosis, N—necrosis, NC—nuclear condensation, A—autophagy, DNAs—DNA synthesis, subG0/G1—apoptotic cell fraction, G0/G1—cell cycle arrest in G0/G1 gap, ROS—oxidative stress, C3/7—caspase 3/7 activity.
